# Novel histone deacetylase inhibitor, CS014, attenuates *in vivo* thrombosis while maintaining hemostasis

**DOI:** 10.1016/j.jtha.2025.11.011

**Published:** 2025-12-11

**Authors:** Livia Stanger, Pooja Yalavarthi, Reheman Adili, Devin Gilmore, Timothy Hoang, Avery Campbell, Paul Krenik, David Gustafsson, Jan Fryklund, Tomas Fex, Nicholas Oakes, Jonas Faijerson Säljö, Björn Dahlöf, Joan Beckman, Michael Holinstat

**Affiliations:** 1 Department of Pharmacology, University of Michigan Medical School, Ann Arbor, MI 48109, USA; 2 Division of Hematology, Oncology, and Transplantation, University of Minnesota, Minneapolis, MN 55455, USA; 3 Emeriti Bio, Mölndal, Sweden; 4 Cereno Scientific, Mölndal, Sweden; 5 Institute of Medicine, University of Gothenburg, Gothenburg, Sweden; 6 Department of Internal Medicine, University of Michigan Medical School, Ann Arbor, MI 48109, USA; 7 Department of Vascular Surgery, University of Michigan Medical School, Ann Arbor, MI 48109, USA

**Keywords:** HDAC, hemostasis, platelet, thrombosis

## Abstract

**Background:**

Epigenetic regulation with histone deacetylase (HDAC) inhibition by valproic acid (VPA) has been used to regulate a number of pathological conditions to date. Recently, VPA was shown to alter production and local release of tissue plasminogen activator and plasminogen activator inhibitor-1 in the blood and to have utility in the regulation of clot formation, resolution, and stability. However, VPA is known to be associated with a rare risk of hepatotoxicity.

**Objective:**

To improve upon VPA, a novel HDAC inhibitor, CS014, was developed with preserved HDAC inhibition and reduced hepatotoxic potential. In this study, we sought to assess the potential of CS014 to function as an antithrombotic drug with a safer profile than VPA.

**Methods:**

CS014 and VPA were assessed for HDAC inhibitory properties as well as 4-ene metabolite production in both *in vivo* and *in vitro* settings. *In vivo* clot formation and bleeding were measured in mice dosed with CS014 or VPA. The direct effects of CS014 and VPA on platelet activity were evaluated *ex vivo*.

**Results:**

CS014 maintained equivalent inhibition of HDAC compared with VPA, without the formation of a key hepatotoxic metabolite, in addition to maintaining the ability to prevent thrombus formation after a vascular injury. Although significant attenuation of platelet accumulation and fibrin formation was observed at the site of injury, CS014 was not observed to alter coagulation or increase bleeding time.

**Conclusions:**

CS014 represents a novel HDAC inhibitor with the potential for reducing hepatotoxicity while maintaining the benefit of preventing injury-induced clotting without increased bleeding diathesis.

## 1 ∣ INTRODUCTION

Thrombotic complications resulting from cardiovascular disease are the leading cause of death globally [1-3]. Currently, strategies to prevent and treat cardiovascular events are primarily accomplished through the use of platelet inhibitors and oral anticoagulants [4,5]. Unfortunately, despite advances in antithrombotic therapies, many patients remain at risk of experiencing a cardiovascular complication, whereas others suffer from an increased risk of bleeding conferred by treatment with antithrombotic medication [3-6]. The shortcomings of current treatment options highlight a continuing challenge in the development of novel therapeutic approaches to limit clot formation without the associated concomitant bleeding. Several novel drugs are in clinical development in both the antiplatelet and anticoagulant fields to try to meet this unmet medical need without impacting bleeding [7-9]. A patient’s level of thrombotic risk is influenced by the competing forces of prothrombotic activity and fibrinolytic capacity. Although current therapies aim to attenuate prothrombotic factors by targeting the coagulation cascade and platelet activity, there is an untapped opportunity to decrease excess thrombotic risk by targeting endogenous fibrinolysis. Decreased tissue plasminogen activator (tPA) or increased plasminogen activator inhibitor-1 (PAI-1), 2 pathophysiological conditions resulting in impaired fibrinolysis, are associated with an increased risk of cardiovascular complications [10-12]. These findings highlight a potential novel approach for targeting thrombosis through restoration of the endogenous fibrinolytic system.

Histone deacetylase (HDAC) inhibitors are metalloenzymes involved in epigenetic modulation [13-16]. Valproic acid (VPA) is a predominantly Class I HDAC inhibitor with some Class IIa inhibition that has long been in clinical use for epilepsy, bipolar disorder, and migraine prevention [17,18]. More recently, *in vitro* studies have demonstrated that VPA and other HDAC inhibitors markedly upregulate gene expression of PLAT [13-15]. Increased expression of *PLAT* alters the balance between tPA and PAI-1, increasing profibrinolytic capacity when needed, which may reduce the risk of thrombosis. Accordingly, pharmacoepidemiologic studies investigating VPA have indicated a reduction in stroke and myocardial infarction, supporting the explanation of increased tPA capacity altering fibrinolytic potential [19-23]. Furthermore, a profibrinolytic shift, measured as an increase in the tPA/PAI-1 ratio, has been observed *in vivo*, both in healthy subjects and in patients given VPA after a myocardial infarction, supporting the use of HDAC inhibition as a means of targeting endogenous fibrinolysis [24,25].

Studies have outlined several potential clinical benefits of HDAC inhibition; however, VPA has been associated with rare hepatotoxic side effects, with a reported incidence of 1 in 20 000 [26,27]. Several mechanisms have been proposed as potential causes of the observed hepatotoxicity [28], including the formation of the metabolite 4-ene-VPA, which enters the mitochondria and is converted into 2,4-diene-VPA [29]. The 2,4-diene metabolite is hepatotoxic, partly due to its conversion into a CoA ester, leading to glutathione consumption [30,31]. Hepatotoxicity is a cause for concern in the clinic, warranting the development of a novel HDAC inhibitor that maintains the beneficial effects observed with VPA without the adverse toxic effects. This study sought to assess CS014, a novel HDAC inhibitor, to determine whether it has preserved HDAC inhibitory potency observed with VPA along with a reduced risk of toxic liver metabolite formation compared to VPA. Further, we sought to confirm whether CS014 exhibits observable antithrombotic effects comparable to those of VPA with a maintained low risk of bleeding.

## 2 ∣ METHODS

A full description of the methods is available in the supplemental materials.

### 2.1 ∣ HDAC inhibitory assay

The HDAC Fluorometric Assay/Drug Discovery Kit was purchased from BioNordika (Enzo). The assay was performed according to the manufacturer protocol. Trichostatin A was used as the model inhibitor, and compounds were tested at 10 concentrations. Raw data were normalized using trichostatin as a control compound. IC50 values for the test compounds were calculated using GraphPad Prism.

### 2.2 ∣ H3 and α-tubulin acetylation

Primary human umbilical vein endothelial cells (HUVECs) were isolated in Lonza culture media as previously described [32]. After treatments, total protein extract was collected, and protein concentration values were obtained with BCA kit (Pierce). Immunoblots were probed with primary antibodies against acetyl-α-tubulin (Lys40), α-tubulin (DM1A), or acetyl-histone H3 (Lys27) (D5E4) and secondary antibodies, followed by visualization using an Azure c600 imager (Biosciences).

### 2.3 ∣ tPA mRNA assessment in HUVECs

Cells were seeded in 6-well plates and incubated with VPA or CS014 before being lysed. The lysates were stored at −80 ° C. Before RNA preparation, lysates were thawed at room temperature and centrifuged. The total RNA was prepared using QIAcube and RNeasy mini-RNA kit plus DNase digestion. Quantification of RNA was performed using the NanoDrop One/OneC Microvolume UV-Vis Spectrophotometer (Thermo Scientific). Levels of tPA mRNA were analyzed using real-time polymerase chain reaction (Applied Biosystems 7500 Fast Real-Time PCR System).

### 2.4 ∣ Assessment of the formation of 2-propyl-4-pentenoic acid (4-ene) (2,4-diene-VPA) *in vitro* and *in vivo*

VPA and CS014 were incubated with human CYP2C9 or pooled human liver microsomes (HLMs). Samples were added to the same volume of ice-cold acetonitrile, vortexed, centrifuged at 10 000*g* and stored at 4 ° C until analysis. Two groups of female rats (Wistar Han) were given a single oral dose of either VPA or CS014 and placed in metabolism cages. The animals were sacrificed 24 hours after collecting urine and taking 1 blood sample from each animal. Formation of 4-ene metabolites was measured in the urine and plasma.

### 2.5 ∣ Bioanalytical method for 4-ene metabolites

Sample supernatants were derivatized to increase the analytical sensitivity. VPA and CS014 were quantified using external standard curves (with the correct degree of deuteration). All 2-propyl-4-pentenoic acids were quantified using external curves of 2-propyl-4-pentenoic acids without deuteration.

### 2.6 ∣ Laser-induced cremaster arteriole thrombosis model

All animal studies were approved by the University of Michigan Institutional Animal Care and Use Committee (PRO00010098). Male wild-type (WT) mice (8-10 weeks of age) were treated via intraperitoneal (IP) injection with saline control, CS014, or VPA twice a day for 5 days. On day 6, mice were anesthetized, and the cremaster was prepared as previously described [33]. The cremaster arterioles were subjected to a laser-induced injury and platelet accumulation, and fibrin formation was monitored by fluorescence intensity (Ablate! photoablation system; 3i Intelligent Imaging Innovations).

### 2.7 ∣ FeCl_3_-induced carotid artery thrombosis assay

Male and female WT mice (8-10 weeks of age) were designated as either recipient or donor mice. Both groups were treated via IP injection with saline control, CS014, or VPA twice a day for 5 days. On day 6, whole blood was drawn from donor mice, and platelets were isolated and fluorescently labeled with calcein acetoxymethyl. Recipient mice were anesthetized by intravenous injection with fluorescently labeled platelets. The right common carotid artery was prepared as previously described [34], and thrombus formation was induced via carotid artery injury. Platelet adhesion, aggregation, and thrombus growth were monitored continuously (Zeiss Axio Examiner Z1) to determine the time to vessel occlusion.

### 2.8 ∣ Human blood collection and platelet preparation

All research involving human subjects was carried out in accordance with the Declaration of Helsinki and was approved by the University of Michigan Institutional Review Board. Written informed consent was obtained prior to blood collection under the approval of the University of Michigan Institutional Review Board (HUM00196782). Washed platelets were resuspended at a physiological concentration of 3 × 10^8^ platelets/mL.

### 2.9 ∣ Platelet aggregation

Washed human platelets were incubated with VPA or CS014 for 10 minutes at 37 ° C. After incubation, platelets were stimulated with an EC_80_ concentration of collagen, and platelet aggregation was measured under stirring conditions (1200 rpm) at 37 °C in a Lumi-Aggregometer (Model 700D; Chrono-Log).

### 2.10.∣ *Ex vivo* microfluidic perfusion flow chamber

Microfluidic perfusion chambers (μ-slide VI 0.1; ibidi) were coated with collagen (Chrono-Log). Whole blood from healthy human donors was incubated with CS014 or VPA, or the equivalent volume of control (saline) for 10 minutes at 37 ° C. Platelets were fluorescently labeled with 2 μM of 3,3′ -dihexyloxacarbocyanine iodide (DiOC_6_; Thermo Fisher Scientific). After recalcification with 5 mM CaCl_2_, the blood perfused at arterial shear (1800/s) [33-35]. Platelet accumulation was quantified by mean fluorescence intensity using SlideBook 6.0 (3i Intelligent Imaging Innovations).

### 2.11 ∣ Flow cytometry

Washed human platelets were treated with CS014, VPA, or control (saline). Platelets were stained with antibodies for active integrin α_IIb_ β_3_, P-selectin, and CD63 and stimulated with convulxin. The surface expression of activation markers was analyzed by measuring fluorescence intensity using a flow cytometer (CytoFLEX; Beckman Coulter).

### 2.12 ∣ Complete blood count

Male and female WT mice (8-10 weeks of age) were treated via IP injection with saline control or CS014 twice a day for 5 days. Before and after treatment, a complete blood count was measured using a complete blood count counter (Hemavet 950FS; Drew Scientific).

### 2.13 ∣ Tail bleeding assay

Male and female WT mice (8-10 weeks of age) were treated via IP injection with saline control, CS014, or VPA twice a day for 5 days. On day 6, mice were anesthetized, and the distal 5 mm of the tail was excised. The tail was immersed in 37 ° C saline solution and bleeding time was assessed.

### 2.14 ∣ Thromboelastography

Male and female WT mice (8-10 weeks of age) were treated via IP injection with saline control, CS014, or VPA twice a day for 5 days. On day 6, citrated whole blood was collected and incubated with control solution or rivaroxaban for 10 minutes. The viscoelastic properties of clot formation were studied under low shear stress using the Thrombelastograph Hemostasis Analyzer (Haemonetics) [36].

### 2.15 ∣ Statistical analysis

Data analysis was performed with Prism 10 (GraphPad Software). Data represent mean ± SEM unless otherwise noted. The specific statistical test used in each experiment is noted in the corresponding figure legend.

## 3 ∣ RESULTS

### 3.1 ∣ CS014

**FIGURE 1:**
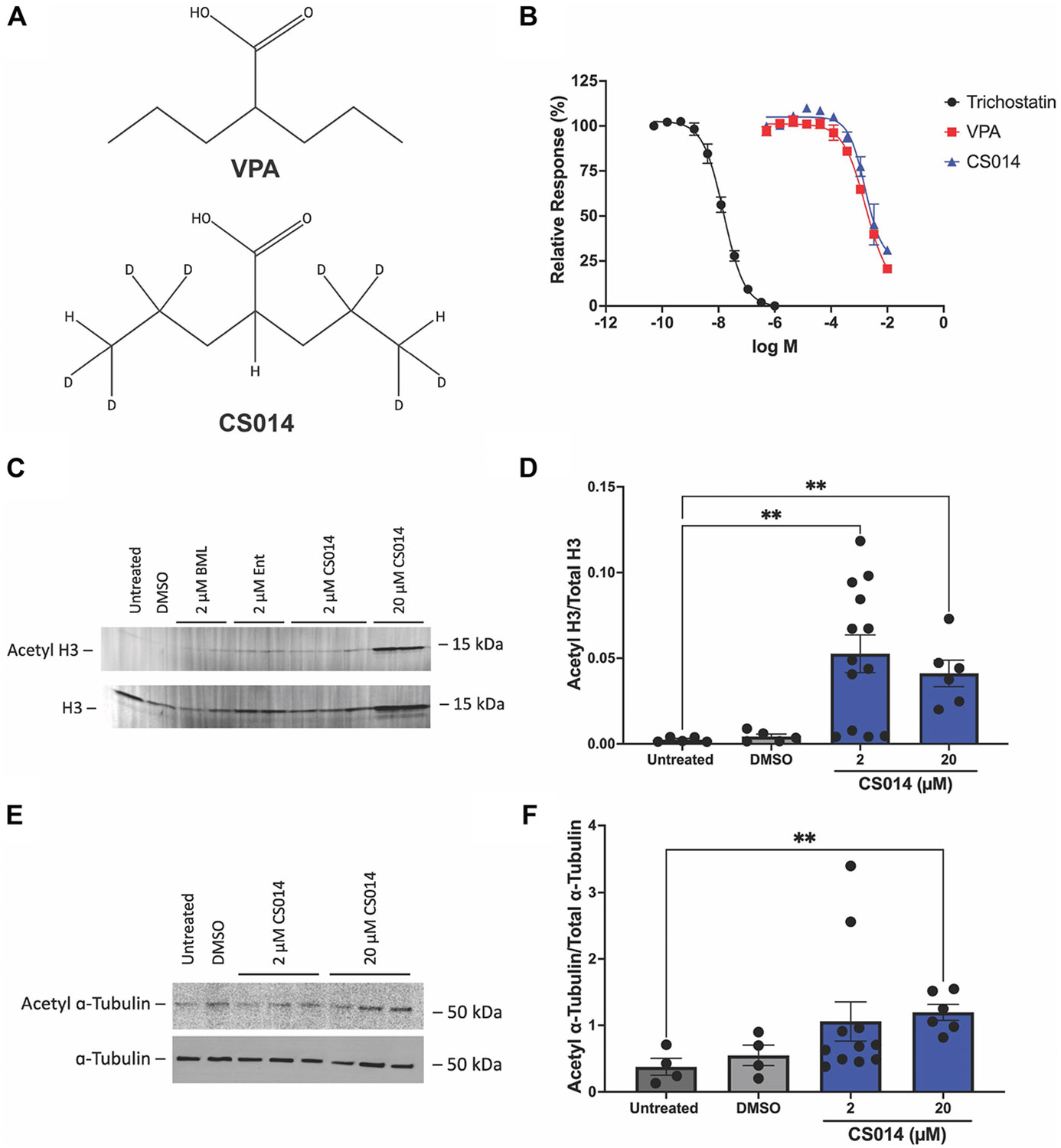
CS014 and VPA inhibit both HDAC3 and HDAC6 activity. (A) Structures of VPA and CS014. (B) Inhibition of HDAC activity expressed as percentage inhibition of control vs log molar concentration of inhibitor. Compounds were tested in concentration-response for >10 concentrations in 1:3 dilution steps with a starting concentration of 10 mM for VPA (squares) and CS014 (triangles). Trichostatin A (circles) was used as a model inhibitor to define the level of no inhibition (100% activity) and full inhibition (0% activity). Data were derived from 3 separate experiments and included technical duplicates for each experiment. The mean IC_50_ values were compared for trichostatin A (15.4 nM, *n* = 3), VPA (1.7 mM, *n* = 3), and CS014 (1.6 mM, *n* = 3) using one-way analysis of variance (anova) with Tukey post-hoc comparison (VPA vs trichostatin A, *P* = .056; CS014 vs trichostatin A, *P* = .019; VPA vs. CS014, *P* = .643), indicating similar potency for VPA and CS014. Data represent mean ± SEM. Total protein lysates from HUVECs were treated for 4 hours with Class IIb inhibitor BML-281 (2 μM), Class I inhibitor entinostat (2 μM), or CS014 followed by separation on a 10% Tris-HCl polyacrylamide gel. (C) Representative immunoblot probed for acetyl-H3 (Lys27) (D5E4) and histone H3 (D1H22). (D) Acetyl-H3 densitometry data normalized to total histone H3. (E) Representative immunoblot probed for acetyl-α-tubulin (Lys40) and α-tubulin (DM1A). (F) Acetyl-α-tubulin densitometry data normalized to total α-tubulin. For densitometry, values are from 3 separate experiments with *n* = 2-3 replicates immunoblotted for each condition. Data represent mean ± SEM. One-way anova with Tukey post-hoc comparison (**P < .01). DMSO, dimethyl sulfoxide; HDAC, histone deacetylase; HUVEC, human umbilical vein endothelial cell; VPA, valproic acid.

We sought to develop a novel HDAC inhibitor with lower hepatotoxic potential than VPA (Figure 1A). The carbon-deuterium (C-D) bond is stronger than the corresponding carbon-hydrogen (C-H) bond and is therefore more difficult for oxidative enzymes to break [37]; therefore, this substitution modifies the metabolic profile of a compound and reduces the formation of potentially toxic metabolites. CS014 is deuterated in both the 4,4′ - and 5,5′ -positions but no other positions (Figure 1A).

### 3.2 ∣ HDAC inhibition by VPA and CS014

The HDAC inhibitory properties of VPA are well established [38]. To determine if CS014 maintains HDAC inhibitory activity equivalent to that of VPA, HDAC activity was measured in the presence of each compound, in addition to trichostatin, a model inhibitor used to define the level of no inhibition and full inhibition. Both CS014 and VPA significantly inhibited HDAC activity, with similar potency measured for the 2 compounds (Figure 1B). Data are presented as inhibition of HDAC activity expressed as percentage inhibition of control vs log concentration. The mean IC_50_ values were not significantly different between VPA (1.7 mM) and CS014 (1.6 mM) (*P* = .643).

### 3.3 ∣ H3 and α-tubulin acetylation

VPA exhibits inhibitory action against Class I HDACs (HDAC1-3 and 8) with some inhibitory function also reported for Class IIa HDACs (HDAC4, 5, 7, and 9) with weaker inhibition of Class IIb (HDAC6 and 10). Therefore, to ascertain if CS014 exhibited activity against Class I and Class II HDACs similar to that of VPA, we performed assessment of acetylation of histone H3, a marker of Class I activity, and tubulin, a marker of Class IIb activity. Consistent with known Class I inhibition, compared with untreated or vehicle-treated cells, CS014 significantly increased acetyl-H3 at both the 2 and 20 μM doses (Figure 1C, D). Cells treated with 2 μM CS014 exhibited similar acetyl-H3 levels to those treated with entinostat, a Class I inhibitor previously found to prevent hypoxia-dependent pulmonary hypertension and prevent fibrosis [39]. Cells treated with the Class IIb inhibitor, BML-281, did not exhibit any H3 acetylation. Next, to assess Class IIb function, we assessed α-tubulin acetylation. Compared to untreated or vehicle-treated cells, cells treated with 20 μM CS014 exhibited significantly increased tubulin acetylation (Figure 1E, F). Combined, these data support the finding that in endothelial cells, CS014 exhibits both Class I and Class II HDAC inhibition. Additionally, these data suggest that at higher concentrations, CS014 also has some Class IIb HDAC6 inhibitory activity.

### 3.4 ∣ tPA mRNA levels

**FIGURE 2:**
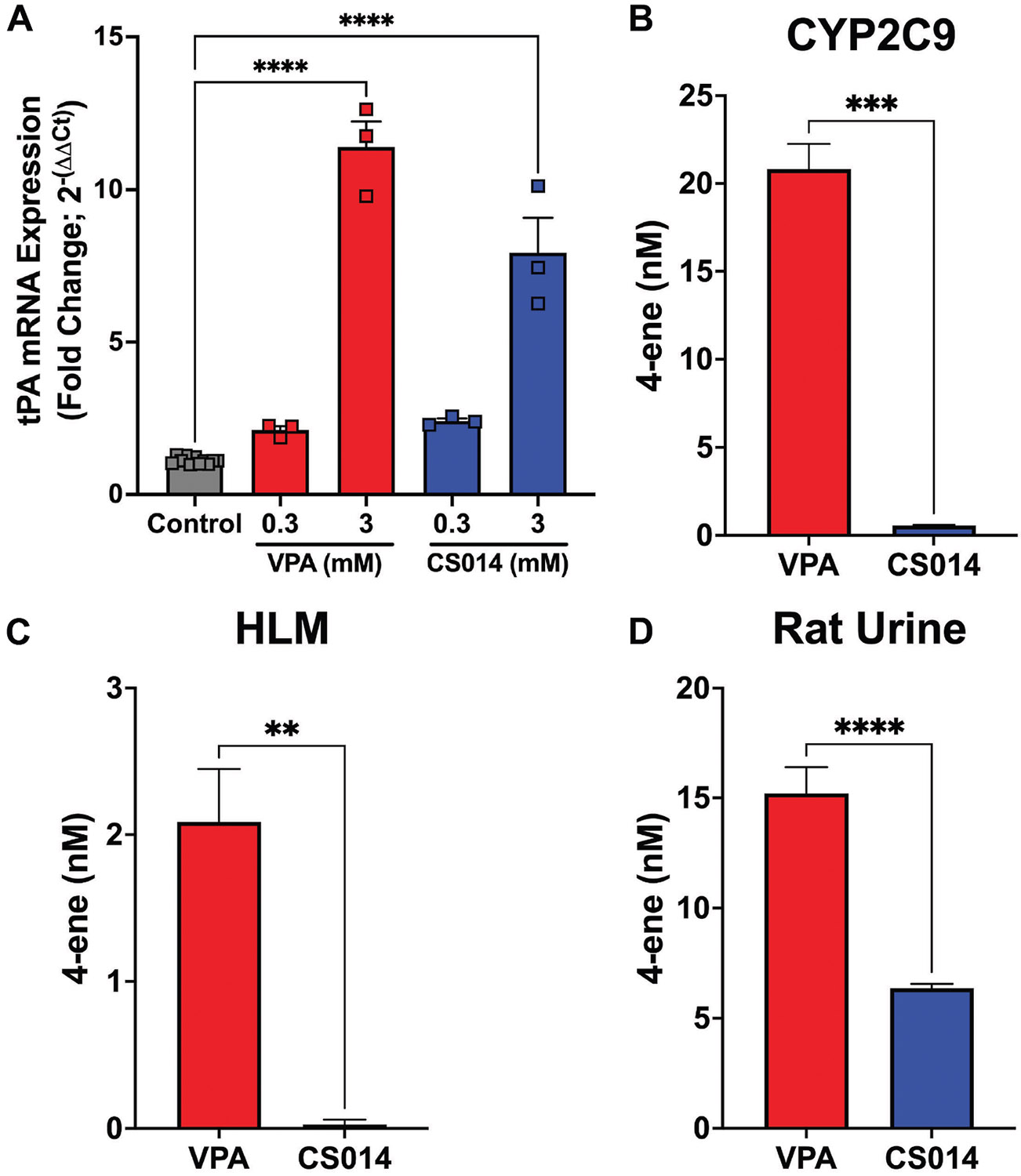
CS014 and VPA increase levels of tPA mRNA, but formation of 4-ene metabolites is higher after incubation with VPA. (A) Mean fold increase in levels of tPA mRNA in HUVECs is higher after incubation for 24 hours with 3 mM of either VPA (*n* = 3; ****P < .0001) or CS014 (*n* = 3; *P* < .0001) than with control (*n* = 11). One-way anova with Tukey post-hoc comparison was performed. Data represent mean ± SEM. (B) In CYP2C9, formation of 4-ene metabolites is significantly lower after incubation with CS014 than with VPA (*n* = 3; ***P < .001). Data represent mean ± SEM. An unpaired, two-tailed *t*-test was performed. (C) Formation of 4-ene metabolites is reduced with CS014 compared with VPA when incubated in HLMs (*n* = 3; **P < .01). Data represent mean ± SEM. An unpaired, two-tailed *t*-test was performed. (D) Female WT rats dosed with VPA have significantly higher levels of 4-ene metabolites measured in the urine 24 hours after dosing compared with CS014-treated rats (*n* = 15; ****P < .0001). Data represent mean ± SEM. An unpaired, two-tailed *t*-test was performed. HLM, human liver microsome; HUVEC, human umbilical vein endothelial cell; tPA, tissue plasminogen activator; VPA, valproic acid; WT, wild-type.

Studies have demonstrated a correlation between HDAC inhibition and epigenetic modification of tPA expression, indicating a potential explanation of how HDAC inhibitors modulate thrombosis [24,40]. A previous study from our group showed that VPA increases tPA mRNA and tPA protein in parallel [14]. To determine whether CS014 has an effect on tPA levels, HUVECs were treated with CS014 or VPA at concentrations of 0.3 and 3 mM, and tPA mRNA levels were assessed. Both VPA and CS014 significantly increased tPA mRNA expression in HUVECs in a concentration-dependent manner (Figure 2A). VPA treatment resulted in a fold increase of tPA mRNA levels of 2.12 ± 0.13 (in the 0.3 mM condition) and 11.39 ± 0.84 (in the 3 mM condition). Treatment of HUVECs with CS014 at concentrations of 0.3 and 3 mM resulted in fold increases of 2.42 ± 0.08 and 7.94 ± 1.14, respectively.

### 3.5 ∣ 4-ene metabolite formation

The formation of 4-ene VPA metabolites is known to have hepatotoxic effects [41,42]. To determine whether the equivalent metabolite is formed by CS014 metabolism, VPA and CS014 were incubated in 3 distinct experimental conditions (*ex vivo* in CYP2C9 and HLMs and *in vivo* in rats), and levels of the corresponding 4-ene metabolites were measured. After incubation of VPA or CS014 with CYP2C9 for 180 minutes, the formation of 4-ene metabolites was 97% lower in the presence of CS014 compared with VPA (Figure 2B). HLMs incubated with VPA and CS014 for 120 minutes had significantly lower concentrations of the 4-ene metabolites than HLMs incubated with CYP2C9. However, the formation of 4-ene metabolites was observed to be 98% lower in the presence of CS014 compared with VPA (Figure 2C). To assess *in vivo* production of the 4-ene metabolites, female rats were dosed via oral gavage with a single dose of either VPA or CS014 and placed in metabolism cages. The animals were sacrificed 24 hours after collecting urine and 1 blood sample. The plasma concentrations of VPA and CS014 were comparable at 24 hours (≈0.8 μM; data not shown). Plasma concentrations of the 4-ene metabolites were undetectable in both the animals given VPA as well as in the animals given CS014 (data not shown). However, in the urine samples, the 4-ene metabolites were detected at significantly higher concentrations in the VPA-treated rats than in the CS014-treated rats (Figure 2D). VPA-treated rats had 15 ± 5 μM 4-ene metabolites in their urine whereas only 6.4 ± 0.8 μM was detected in urine from CS014-treated rats, representing 0.99% and 0.53% of the parent compounds in urine, respectively (*P* < .0001, *t*-test).

### 3.6 ∣ CS014 effects on clotting in small vessels

**FIGURE 3:**
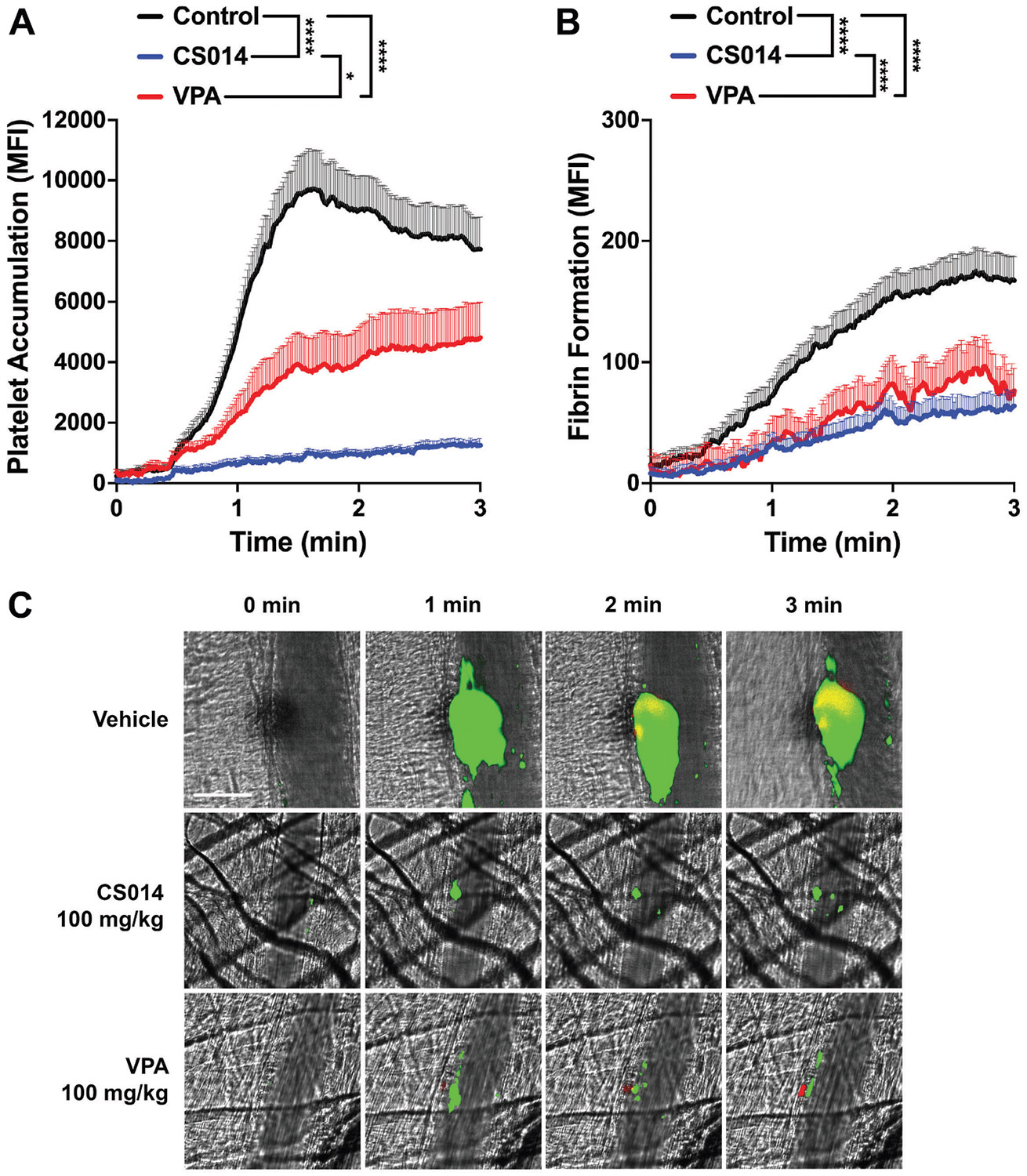
Both CS014 and VPA inhibit clotting in small vessels. Wild-type mice were treated twice daily for 5 days with saline control, 100 mg/kg VPA, or 100 mg/kg CS014 via intraperitoneal injection. The cremaster arterioles of anesthetized mice were injured using a focused laser-induced injury assay. Platelet accumulation (A) in control mice (black; 1 mouse, 9 injuries), VPA-treated mice (red; 2 mice, 7 injuries), and CS014-treated mice (blue; 2 mice, 14 injuries) and fibrin formation (B) in control mice (black; 1 mouse, 9 injuries), VPA-treated mice (red; 1 mouse, 3 injuries), and CS014-treated mice (blue; 2 mice, 9 injuries) at the site of injury were measured in real-time using *in vivo* intravital microscopy. Data represent mean ± SEM (SEM is shown only for control condition). (C) Representative images of data presented in A and B showing platelet plug formation (green) and fibrin accumulation (red). Scale bar represents 100 μm. MFI, mean fluorescence intensity; VPA, valproic acid.

To determine the effects of CS014 on thrombosis, male WT mice were treated via IP injection twice daily for 5 consecutive days with either saline control, 100 mg/kg VPA, or 100 mg/kg CS014. On day 6, thrombus formation was assessed after vascular injury using real-time intravital microscopy. A laser-induced injury was made to the cremaster arteriole, and platelet and fibrin accumulation were monitored at the site of injury [33,43]. Mice treated with VPA or CS014 showed lower platelet accumulation than control-treated mice (Figure 3A). Treatment with VPA or CS014 also resulted in reduced fibrin formation at the site of injury (Figure 3B). Platelet formation was visualized in real-time in green, with fibrin formation in red (Figure 3C). We observed a larger degree in attenuation of platelet accumulation with CS014 than with VPA.

### 3.7 ∣ CS014 delays occlusive thrombosis in large vessels

**FIGURE 4:**
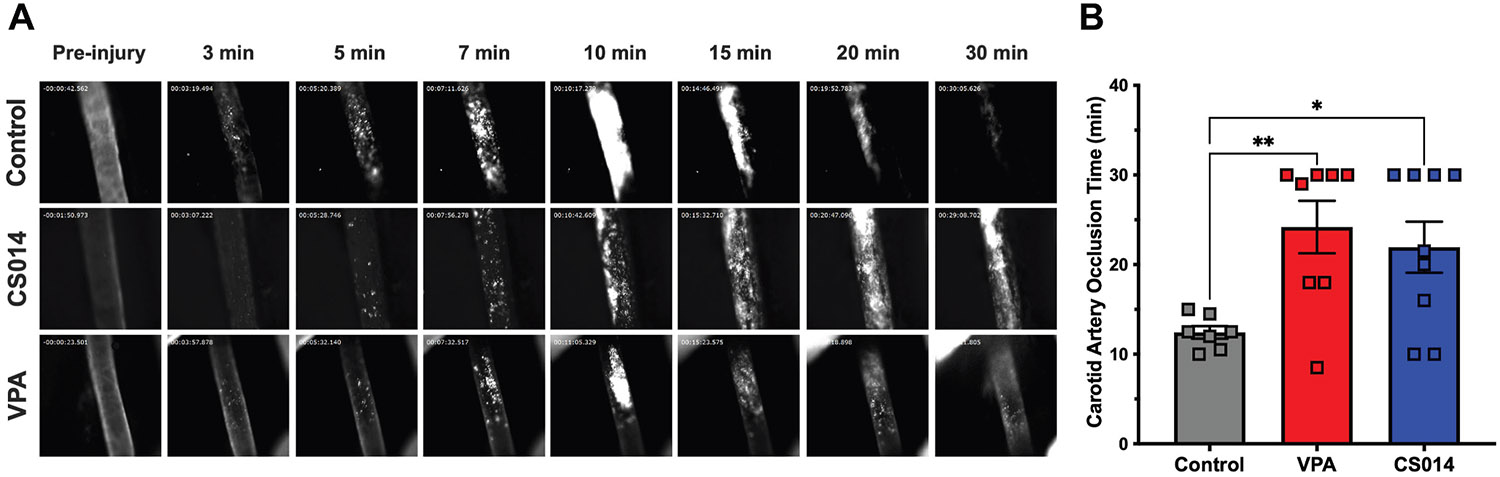
Both CS014 and VPA attenuate thrombosis in a large vessel. Wild-type mice were administered saline control, 100 mg/kg VPA, or 100 mg/kg CS014 via intraperitoneal injection twice daily for 5 days. After treatment, a carotid artery injury was induced with a FeCl_3_-soaked patch, and thrombosis in the vessel was monitored by intravital microscopy for 30 minutes. (A) Representative images show fluorescently labeled platelets visualized at the site of injury. (B) Experiments were terminated 30 minutes after injury, and animals whose vessels did not occlude within the 30-minute timeframe were recorded with an occlusion time of 30 minutes (*n* = 7-10). Ordinary one-way anova with Dunnett’s multiple comparisons (*P < .05; ***P < .001). VPA, valproic acid.

To determine if HDAC inhibition by VPA or CS014 can attenuate an occlusive thrombotic event in large arteries, thrombus formation was assessed using the carotid artery injury model. Male and female WT mice were treated twice daily for 5 days with saline control, VPA (100 mg/kg), or CS014 (100 mg/kg) via IP injection. On day 6, the carotid artery was exposed, and a severe vessel injury was induced by treating with a 1-mm^2^ filter paper immersed in 10% FeCl_3_. After 2 minutes, the FeCl3-soaked filter paper was removed, and adhesion of fluorescently labeled platelets was monitored using intravital microscopy (Figure 4A). The time to vessel occlusion after injury was measured for up to 30 minutes; if the vessel failed to form an occlusive thrombus by then, an occlusion time of 30 minutes was recorded. Whereas the average occlusion time in control-treated mice was 12.43 ± 0.70 minutes, full vessel occlusion time in animals treated with VPA or CS014 was significantly delayed, averaging 24.19 ± 2.93 and 21.94 ± 2.85 minutes, respectively (Figure 4B).

### 3.8 ∣ Direct platelet effects of CS014 and VPA

**FIGURE 5:**
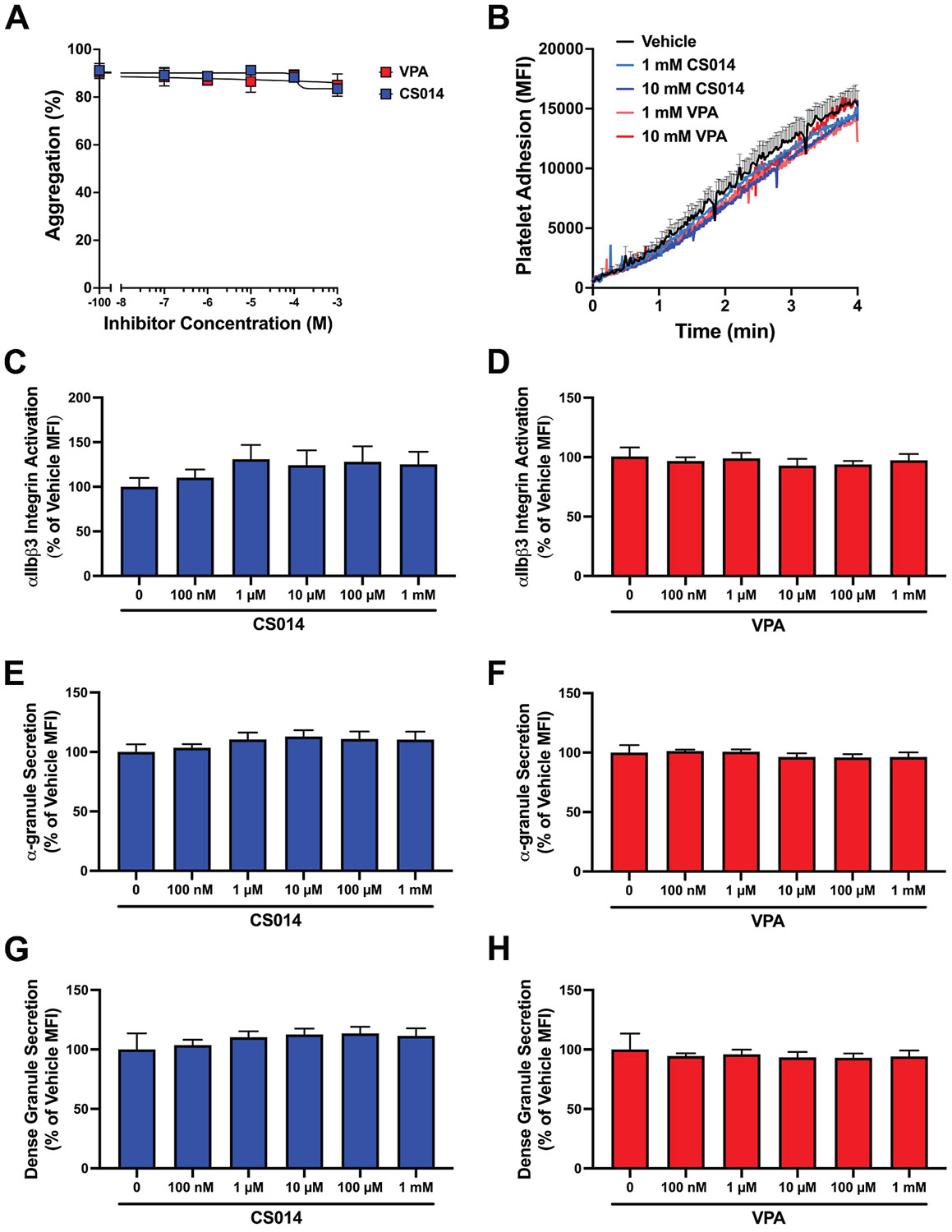
Neither CS014 nor valproic acid (VPA) directly regulate platelet function. (A) Aggregation of washed human platelets treated with increasing concentrations of VPA or CS014 for 10 minutes prior to stimulation with an EC_80_ concentration of collagen (0.0625-0.25 μg/mL). Percentage of platelet aggregation 10 minutes after stimulation was assessed. Data represent mean ± SEM (*n* = 4). (B) Citrated human whole blood was treated with either 1 mM or 10 mM VPA or CS014 for 10 minutes and stained with DiOC_6_. After recalcification, the stained blood was perfused through a collagen-coated chamber at arterial shear (1800/s). Platelet adhesion was quantified using mean fluorescence intensity (MFI). Two-way anova was used to compare each condition to the vehicle control (saline). Data represent mean ± SEM (*n* = 5). Washed human platelets were treated with VPA or CS014 for 10 minutes prior to stimulation with 25 ng/mL of convulxin. Markers of platelet activation were assessed at increasing concentrations of VPA or CS014 from 0 to 1 mM. No significant difference was observed in activation of integrin αIIb β3 (C, D), α-granule secretion (measured by P-selectin expression) (E, F), and dense granule secretion (measured by CD63 expression) (G, H). Data represent mean ± SEM (*n* = 5). One-way anova with Dunnett’s multiple comparisons test.

Since the maximum size of the clot formed in the presence of HDAC inhibitors was reduced and fibrin formation was decreased, it was important to determine if this inhibition was due to the actions of the compounds as HDAC inhibitors or through a direct action on the platelets independent of HDAC inhibition. To determine whether either compound had a direct effect on the activity of human platelets, washed human platelets were isolated from healthy donors, and collagen-induced platelet aggregation was assessed in the presence of VPA or CS014. Platelets were treated with increasing concentrations of VPA or CS014 up to 1 mM; no effect on platelet aggregation was observed with either VPA or CS014 (Figure 5A). To investigate whether any inhibitory effects of VPA or CS014 are observed in a whole blood condition, citrated human whole blood was treated with vehicle control (saline), VPA (1 mM or 10 mM), or CS014 (1 mM or 10 mM). Platelets were labeled using DiOC_6_, and the blood was perfused through a collagen-coated chamber under arterial shear conditions. Platelet adhesion to the collagen-coated chamber was measured; no antiplatelet effect of either VPA or CS014 was detected (Figure 5B). To further assess the effects of the HDAC inhibitors on platelet activation after incubation with VPA or CS014, the levels of 3 markers were assessed by flow cytometry. Activation of the treated platelets via GPVI receptor clustering was induced by the addition of 25 ng/mL convulxin. Neither VPA nor CS014 impacted the activation of the integrin α_IIb_ β_3_ (Figure 5C, D). Similarly, neither compound affected platelet α-granule secretion in response to stimulation with convulxin (as measured by surface expression of P-selectin, a marker of α-granule secretion) (Figure 5E, F). Finally, levels of CD63, a marker of platelet dense granule secretion, were also unchanged in the presence of VPA or CS014 (Figure 5G, H).

### 3.9 ∣ CS014 does not affect platelet count

**FIGURE 6:**
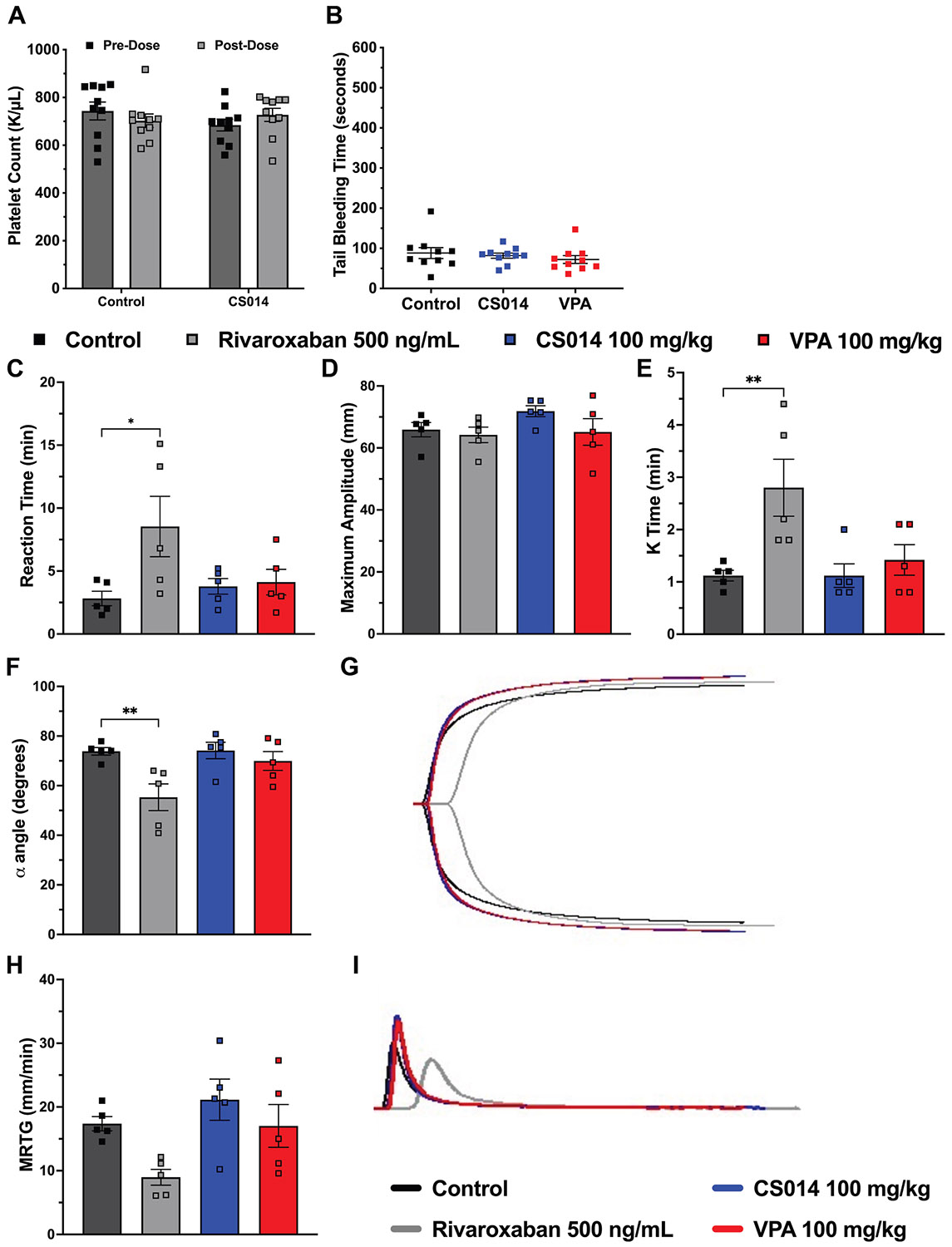
CS014 and VPA do not affect platelet count, bleeding risk or coagulation. (A) WT mice were treated with saline control or 100 mg/kg CS014, and platelet count was measured before and after the 5-day twice-daily dosing period. Data represent mean ± SEM. Two-way anova with Fisher’s least significant difference test for multiple comparisons (*n* = 10). (B) WT mice were treated with saline control, 100 mg/kg VPA, or 100 mg/kg CS014 twice daily for 5 days. Bleeding risk was assessed using the tail bleeding assay. Data represent mean ± SEM. Ordinary one-way anova with Dunnett’s multiple comparisons test (*n* = 10). (C–I) WT mice were dosed twice daily with saline control, 100 mg/kg CS014, or 100 mg/kg VPA for 5 days. After dosing, whole blood was drawn from the treated mice and pretreated with either dimethyl sulfoxide control or 500 ng/mL rivaroxaban for 10 minutes. Parameters related to clotting factors and clotting time were assessed by thromboelastography (TEG). (C) Reaction (R) time, time to initiation of fibrin clot formation (min). (D) Maximum amplitude, the maximum strength of the clot (mm). (E) K time, the time until the clot reaches a strength of 20 mm (min). (F) Alpha (α) angle, the rate of clot formation (degrees). Data represent mean ± SEM (n = 5). Ordinary one-way anova with Dunnett’s correction (*P < .05; **P < .01). (G) Representative tracing of the coagulation parameters measured in C–F. (H) Maximum rate of thrombin generation (MRTG) was measured using a velocity curve graphing strength over time (mm/min). (I) Representative tracing of the thrombin generation parameter quantified in H. Data represent mean ± SEM (*n* = 5). Ordinary one-way anova with Dunnett’s correction. VPA, valproic acid; WT, wild-type.

Due to the observed antithrombotic effects and the lack of direct antiplatelet effect observed in the presence of CS014, we sought to determine whether CS014 was impacting the total platelet count. WT mice were treated with 100 mg/kg CS014 or saline control twice a day for 5 days. Before and after administration, a complete blood count was measured to quantify platelet count. There was no difference in platelet count before and after dosing in either treatment group (Figure 6A). Additionally, no other blood cells were affected by the treatment (Table).

### 3.10 ∣ CS014 and VPA do not alter hemostatic potential

Treatment with many antithrombotic drugs that target platelets to prevent thrombosis results in increased bleeding diathesis, which carries its own risk of morbidity and mortality [4,44]. To determine if the antithrombotic effect observed due to HDAC inhibition contributes to an increased risk for bleeding, WT mice were treated via IP injection with either 100 mg/kg CS014 or 100 mg/kg VPA twice a day for 5 days and assessed for their hemostatic response. After administration of either compound, the mouse tail vein bleeding assay was performed whereby 5 mm of the distal tail of the mouse was resected, and the amount of time between resection and the cessation of bleeding was recorded. In control-treated animals, approximately 90 seconds lapsed after resection of the tail before the tail clotted and stopped bleeding (Figure 6B). VPA treatment resulted in a time to cessation of bleeding that averaged approximately 70 seconds, but this was not significantly different from control animals. CS014-treated animals did not have significantly different bleeding times than control animals, averaging approximately 80 seconds until cessation of bleeding.

To determine if HDAC inhibition altered coagulation function, coagulation was assessed in blood drawn from mice treated with either saline control, 100 mg/kg CS014, or 100 mg/kg VPA via IP injection twice a day for 5 days. Drawn whole blood was incubated with dimethyl sulfoxide control or 500 ng/mL rivaroxaban (a factor Xa inhibitor) for 10 minutes prior to assessment of coagulation parameters using thromboelastography (TEG). TEG assesses activation of the contact pathway in the blood and measures clot formation and strength over time. Neither CS014 nor VPA treatment resulted in a delay in reaction time (Figure 6C), maximal amplitude (Figure 6D), K time (Figure 6E), or α angle (Figure 6F) compared with the control condition. Rivaroxaban is a FXa inhibitor that impairs the coagulation pathway and has been shown to decrease reaction time in TEG and rotational TEG assays [45]. The addition of rivaroxaban confirms the assay accurately measured the activity of the coagulation pathway, as rivaroxaban-treated blood showed a significant delay in reaction time compared with control conditions. Finally, the maximum rate of thrombin generation was measured and similarly showed no differences between the CS014- or VPA-treated groups when compared with the control (Figure 6H). Representative tracings show no difference between control-, CS014-, and VPA-treated mice, while addition of rivaroxaban significantly altered the resulting curve (Figure 6G, I).

## 4 ∣ DISCUSSION

Arterial and venous thromboembolism continue to be life-threatening conditions, and millions of people are treated annually with antithrombotic drugs. Although antithrombotic drugs provide a net benefit, many patients exhibit significant bleeding events as a consequence of this intervention [3-5]. Novel antithrombotic drugs with reduced bleeding risk are targets for the development of new therapeutics to prevent or treat thromboembolic events. VPA was serendipitously discovered as an antiepileptic agent without knowledge of its HDAC inhibitory properties [46].

Pharmacoepidemiologic cardiovascular data from VPA-treated patients provided the first indirect evidence that this approach may have additional utility in the prevention of stroke and myocardial infarction [19-23], possibly due to the observed increase in local release of tPA and reduction of PAI-1 in circulation [13,14] or other epigenetic effects on hemostasis [47,48]. Our data indicate that VPA and CS014 increase levels of tPA mRNA in HUVECs, which we previously reported (Figure 2A) [40]. A consequence of altered tPA and PAI-1 levels in the blood should be decreased potential of fibrin formation or increased potential of fibrin resolution. Supporting previous studies that suggested HDAC inhibition altered tPA and PAI-1, the *in vivo* cremaster thrombosis assay showed a decrease in fibrin formation and reduced clot stability after a vascular injury.

There are 18 different HDACs that are subcategorized into 4 groups: Class I (HDAC1-3 and 8), Class IIa (HDAC4, 5, and 9), Class IIb (HDAC6 and 10), Class III (SIRT1-7), and Class IV (HDAC11). Based on H3 acetylation, CS014 exerts inhibition against Class I HDACs (1-3 and 8). Interestingly, endothelial cells treated with higher concentrations of CS014 exhibit a modest increase in α-tubulin acetylation, consistent with Class IIb HDAC6 inhibition or potentially NAD+-dependent sirtuin 2 (SIRT2) activity; however, immunoblotting results are not specific to SIRT inhibition. VPA is not considered an HDAC6 inhibitor [49]. For all HDAC inhibitors, in HeLa cells, CS014 had an inhibitor level of ~2 mM (Figure 1).

CS014 was observed to be well-tolerated *in vivo* as denoted by no observable change in weight or appearance of the animals over the 5-day dosing period (data not shown). Additionally, the animals exhibited significant protection from platelet activation and accumulation, resulting in reduced thrombotic potential in several *in vivo* thrombosis assays, including the cremaster thrombosis assay and carotid artery thrombosis assay (Figures 3 and 4). The protection from injury-induced thrombosis was observed in small and large vessels on the arterial side of the vascular bed. These observations support the potential of CS014 as an antithrombotic agent with significant *in vivo* utility across the vasculature. Although HDAC inhibition has previously been suggested to be antithrombotic by our group and others [13-15,24,25,40], this is one of the first demonstrations of *in vivo* antithrombotic effects by HDAC inhibition, highlighting HDAC inhibitors as a potential new class of antithrombotic drugs [40].

Apart from HDAC6, which participates in cortactin and microtubule platelet activities during maturation, platelets do not have robust HDAC expression [50-52]. Therefore, given the proposed mechanism of the antithrombotic effects of CS014 through Class I HDAC inhibition, we did not anticipate significant changes in platelet function. Indeed, the platelet response to VPA and CS014 was confirmed in *ex vivo* assays using aggregation, flow cytometry, and perfusion flow chamber, all of which indicated no direct effect on platelet activity (Figure 5). Of note, in our endothelial assays, CS014 did exhibit a concentration-dependent increase in α-tubulin acetylation (Figure 1). Platelets deacetylate tubulin through HDAC6 in an agonist concentration- and time-dependent manner, likely through GPVI [51]. However, *HDAC6* knockout mouse platelets maintain aggregation and spreading, albeit with delayed kinetics [52]. Interestingly, early exposure of cold-stored platelets to HDAC6 inhibition improved hemostatic function without altering platelet function [53]. This suggests, along with our studies, that novel platelet-independent antithrombotic effects of HDAC inhibitors, such as CS014, could provide a new tool in dual antithrombotic therapy. Implementing a combination of therapeutics without solely relying on decreasing platelet activity could be beneficial in avoiding a bleeding crisis [54,55].

Bleeding is always a concern with any intervention that alters coagulation parameters, fibrin, or platelet function. To address these concerns, we measured platelet count pre- and postdosing with CS014, and we assessed CS014 by tail bleeding time as well as TEG. No difference in platelet count was observed in mice treated with CS014 (Figure 6A). Tail bleeding time was not significantly different in mice treated with CS014, VPA, or control (Figure 6B). In TEG, no observable difference was seen in any parameter measured after CS014 or VPA treatment (Figure 6C-I). Rivaroxaban did, however, significantly delay reaction time during TEG, as has been previously reported for FXa inhibitors [56]. Together, results from the TEG and tail bleeding assays suggest that bleeding may not be a risk factor when attenuating thrombosis using HDAC inhibitors such as VPA and CS014.

Targeting thrombosis through inhibition of HDACs would allow us to utilize the endogenous fibrinolytic system to treat patients with thrombotic disorders. Although this approach to treatment was only recently proposed, the effects of HDAC inhibition on thrombus formation is supported by the literature [14,15,40]. Currently, there is a safety concern related to the side effects of VPA, particularly with regard to the drug’s rare hepatotoxicity. Compared with VPA, CS014 was shown in the present study to maintain its Class I HDAC inhibition *in vivo* and increase levels of tPA mRNA while reducing hepatotoxic potential based on marked reduction in 4-ene metabolite formation. Although toxicity studies have not been conducted to date, the reduction in 4-ene metabolite formation is encouraging as we expect lower levels of the 2,4-diene metabolite to alleviate symptoms of hepatotoxicity [57,58]. Importantly, the *in vitro* and *in vivo* data presented here characterize CS014 as a potential novel, efficacious, and better tolerated HDAC inhibitor for the treatment of cardiovascular diseases such as thrombosis, myocardial infarction, and stroke, thus representing a new path toward protection for patients with increased thrombotic risk.

## Supplementary Material

1

The online version contains supplementary material available at https://doi.org/10.1016/j.jtha.2025.11.011.

## Figures and Tables

**TABLE:** Complete blood count of mice pre- and postinjection with CS014 or vehicle control.

CBC (*n* = 10)	Vehicle		CS014	
	Preinjection	Postinjection	Preinjection	Postinjection
WBC (×10^3^/μL)	9.9 ± 2.45	10.78 ± 3.42	10.87 ± 2.65	12.54 ± 5.11
RBC (×10^3^/μL)	10.11 ± 0.77	10.13 ± 0.68	9.82 ± 1.09	9.81 ± 1.19
PLT (×10^3^/μL)	743.3 ± 118.13	702.4 ± 89.58	684.4 ± 78.67	727.2 ± 87.04
MPV (fL)	4.54 ± 0.13	4.57 ± 0.20	4.67 ± 0.26	4.63 ± 0.50

CBC, complete blood count; MPV, mean platelet volume; PLT, platelet; RBC, red blood cell; WBC, white blood cell.
