# Supplementary material for: Novel histone deacetylase inhibitor, CS014, attenuates in vivo thrombosis while maintaining hemostasis
Source: J Thromb Haemost. Author manuscript; Available in PMC 2026 Jun 30. (PMC13318190; doi:10.1016/j.jtha.2025.11.011)
Supplement: 1 [file NIHMS2187474-supplement-1.docx]

**Supplemental Methods**

**Reagents**

CS014 (semi-sodium salt of octadeutero-2-propylpentanoic acid, Red Glead Discovery Lund, Sweden), NADPH (Combi Blocks), K_2_HPO_4_, MgCl_2_, 3-Nitrophenylhydrazibe hydrochloride, valproic acid sodium salt, N-(3-Dimethylaminopropyl)-N’-ethylcarbodiimide, and 2-propyl-4-pentenoic acid (Sigma-Aldrich). Acetonitrile**,** ammonium formate, formic acid, and methanol (Fischer Scientific).

**HDAC inhibitory assay**

HDAC Fluorometric Assay/Drug Discovery Kit was purchased from BioNordika (Enzo). Human CYP2C9 was from Cypex (UK) and pooled human microsomes from BioIVT. HUVEC cells purchased from Lonza were pre-cultured in a 75cm^2^ culture flask in Basal Medium (Lonza) with supplements (Lonza). RLT buffer, RNeasy mini-RNA kit + DNase digest were from Qiagen. All cDNA and TaqMan reagents were obtained from Applied Biosystems. The assay was run according to the manufacturer protocol. Briefly, samples were incubated for 20-30 minutes in a 96-well plate with substrate, which comprises an acetylated side chain, and HeLa extract (source of HDAC activity). Developer was added and samples were incubated for 15 min. The signal was read on a fluorometric plate reader (Pherastar FS), ex 360 nm/em 440 nm. Trichostatin A was used as the model inhibitor and was tested in a concentration-response manner, starting at 1 µM. Compounds were tested at 10 concentrations using 1/3-dilutions with a starting concentration of 10 mM for VPA and CS014. Raw data were normalized using Trichostatin as a control compound. IC_50_ values for the test compounds were calculated using GraphPad Prism 10, using Curve fitting and non-linear regression, four parameter variable slope.

**H3 and α-tubulin acetylation**

Primary human umbilical vein endothelial cells (HUVEC) were isolated as previously described in Lonza culture media^1^. Cells were grown until 95 % confluent in 100 mm dish, then changed to Lonza media supplemented with 0.1 % FBS. All cells were between passages 2-5 for experiments. For treatments, HUVEC grown in 0.1 % FBS medium were incubated with CS014 (2-20 µM), BML-281 (2 µM, Enzo GR361), or Entinostat (2 µM, MS-275, Selleckchem) for 4 h. After treatments, total protein extract was collected using 20 mM Tris-HCl (pH 7.5), 150 mM NaCl, 1 mM Na2EDTA, 1 mM EGTA, 1 % Triton, 2.5 mM sodium pyrophosphate, 1 mM beta-glycerophosphate, 1 mM Na3VO4, 1 µg/ml leupeptin buffer (Cell Signaling Technologies) supplemented with 1 mM PMSF and phosphatase inhibitor cocktail (Millipore Sigma). Protein concentration values were obtained with BCA kit (Pierce). The lysates were run on 10 % Mini-PROTEAN^®^ TGX™ Precast Protein Gels (BioRad), transferred to PVDF membrane and stained for total protein using fluorescent AzureRed Protein Stain (Azure Biosystems). After blocking (Odyssey blocking buffer, Li-Cor), immunoblots were probed with primary antibodies for acetyl-α-tubulin (Lys40; D20G3, cat. 5335) and α-tubulin (DM1A, cat. 3873) or Anti-Histone H3 (acetyl K27; EP16602, ab177178) and secondary antibodies followed by visualization using an Azure c600 imager (Azure, Biosciences). Membranes were stripped of primary and secondary antibodies using New Blot™ IR Stripping Buffer (Licor), blocked, and reprobed with Histone H3 (D1H2; cat. 4499). Experiments were performed at least 3 times with densitometry quantification completed using Fiji software (NIH).

**tPA mRNA assessment in HUVEC cells**

Cells were seeded in 6-well plates, 0.5 x 10^6^ cells/well in 2 mL basal medium with supplements and incubated (37°C/5 % CO_2_/95 % humidified incubator) for 24 hours. Media was removed and replaced with fresh media (control wells) and media + VPA or CS014 was added to confluent cells and incubated for another 24 hours. Media was removed and cells were lysed using 400 µL RLT buffer (40 µL 1 mM DTT was added per mL of RLT buffer). The lysates were transferred to a 1.5 mL Eppendorf tube and stored at -80°C.

Before RNA preparation lysates were thawed at room temperature and centrifuged at 12,000 *g* for 3 minutes, 350 µL were transferred to 2 mL Eppendorf tubes. The total RNA was prepared using Qiacube and RNeasy mini-RNA kit + DNase digest. RNA was eluted in 30 µL of water. Quantification of RNA was done using Nanodrop (Thermo Scientific, NanoDrop™ One/OneC Microvolume UV-Vis Spectrophotometer). RNA (2 μg) was used for each reverse transcription reaction to generate cDNA (cDNA reverse transcription kit). Levels of tPA mRNA were analyzed with real-time RT PCR (Applied Biosystems 7500 Fast Real Time PCR Systems), using TaqMan fast advanced Master mix (Applied Biosystems). The real time PCR reaction was set up for HPRT (primer/probe Hs99999909_m1) and tPA (primer/probe Hs00263492_m1). 80 ng cDNA was used for each reaction. The average of two Ct (cycle threshold) values for the housekeeping gene (HPRT) and for tPA was calculated using the ΔΔCt method. Each Ct value is the average of three replicates in the TaqMan assay. ΔCt was calculated (ΔCt = Ct tPA/HPRT - Ct HPRT), for both tPA and HPRT. Then the difference between ΔCt tPA and ΔCt HPRT was calculated to generate the double Delta Ct value (ΔΔCt). The value of 2^-(ΔΔCt)^ was calculated to get the expression fold change. For each gene and concentration (Ct) two Ct-values (3 replicates for each Ct) were obtained. The average of these values was calculated and used to generate the ΔCt-value. ΔCt = Ct tPA - Ct HPRT; ΔΔCt = ΔCt tPA - average control ΔCt HPRT. The value of 2^-(ΔΔCt)^ was calculated to get the expression fold change.

**Assessment of the formation of 2-propyl-4-pentenoic acid (4-ene) (2,4-diene-VPA) *in vitro* and *in vivo***

Recombinant human CYP2C9: VPA and CS014 (1 mM) were incubated for 180 minutes with human CYP2C9 (0.2 pmol/μL) in potassium phosphate buffer (100 mM, pH 7.4) containing magnesium chloride (5 mM) and NADPH (2 mM). Samples were withdrawn from incubation before addition of NADPH at time 0 and 180 minutes after addition of NADPH. Samples were added to the same volume of ice-cold acetonitrile, vortexed, centrifuged at 10,000 *g*, and stored at 4^o^C until analysis.

Human liver microsomes: VPA and CS014 (1 mM) were incubated for 120 minutes with pooled human microsomes (0.5 mg/mL) in potassium phosphate buffer (100 mM, pH 7.4) containing magnesium chloride (5 mM) and NADPH (2 mM). Samples were withdrawn from incubation before addition of NADPH at time 0- and 120-minutes following NADPH addition. Samples were added to the same volume of ice-cold acetonitrile, vortexed, centrifuged at 10,000 *g*, and stored at 4^o^C until analysis.

Rat: Two groups of female rats (Wistar Han, n = 15/group, RISE, Ethical License No. S7-15) were given a single oral dose of either VPA (100 mg/kg or 694 µmol/kg) or CS014 (106 mg/kg or 694 µmol/kg) in equal molar concentrations in H_2_O and placed in metabolism cages. During the initial 0 – 4 hour period, the animals were fasted but had free access to water. Thereafter, the animals had free access to both water and feed. The animals were sacrificed at 24 hours after collecting urine and taking one blood sample from each animal. Formation of 4-ene metabolites was measured in the urine collected during the entire 24 hours period. Plasma concentrations of VPA, CS014, and respective 4-ene metabolites were measured in the blood samples taken at 24 hours. The rat study did not use derivatization to increase sensitivity as described below.

**Bioanalytical method for 4-ene metabolites**

To increase the analytical sensitivity, the sample supernatants were derivatized according to the following protocol: 50 µL sample or standard was mixed with 20 µL of N-(3-Dimethylaminopropyl)-N’-ethylcarbodiimide (120 mM) and 50 µL of 3-Nitrophenylhydrazibe hydrochloride (200 mM), both in water. The samples were sonicated for 30 minutes at 40°C. The reaction was quenched by adding 200 µL of 0.1 % formic acid in water. The samples were then diluted to the appropriate concentration with acetonitrile (50 %) in water and formic acid (0.1 %) and analyzed by UPLC (Agilent 1290 Infinity II LC system, Agilent 6495B Triple Quadropole LC/MS with iFunnel Technology using a Zorbax Eclips Plus C18 RRHD 2,1 x 50 mm 1.8 µm column).

VPA and CS014 were quantified using external standard curves (with the correct degree of deuteration). All 2-propyl-4-pentenoic acids were quantified using external curves of 2-propyl-4-pentenoic acids without deuteration.

**Laser-induced cremaster arteriole thrombosis model**

All animal studies were approved by the University of Michigan IACUC committee (Protocol #: PRO00010098). The cremaster arteriole thrombosis model requires male mice since female mice do not have a cremaster; thus, only male mice are used in this assay. Male C57BL/6 wild-type (WT) mice (8 – 10 weeks of age) were treated via intraperitoneal (IP) injection with saline control, CS014 (100 mg/kg), or VPA (100 mg/kg) twice a day for 5 days. On day 6, mice were anesthetized by IP injection of ketamine/xylazine and a tracheal tube was inserted to facilitate breathing. A jugular vein catheter was established to deliver antibodies intended to fluorescently label circulating platelets (DyLight 488 anti-GPIbβ, Emfret Analytics Eibelstadt, Germany) and formed fibrin (anti-mouse fibrin antibody, a kind gift from Dr R. Camire, Children’s Hospital of Philadelphia, was labeled using an Alexa Fluor 647 labeling kit from Thermo Fisher, Waltham, MA). The cremaster muscle was surgically prepared under a dissecting microscope and superfused with preheated bicarbonate saline buffer (132 mM NaCl, 4.7 mM KCl, 1.2 mM MgSO_4_, 2 mM CaCl_2_, 18 mM NaHCO_3_) throughout the experiment. The cremaster muscle arterioles (30 – 50 μm in diameter) were visualized in real-time using a 63X water-immersion objective, with a Zeiss Axio Examiner Z1 multichannel fluorescent microscope. A precise injury was created in the wall of the arteriole, induced using a laser ablation system (Ablate! Photo-ablation system; Intelligent Imaging Innovations, Denver, CO) in order to generate thrombus formation^2^. Images were acquired in real-time with a high-speed sCMOS camera. Multiple laser injuries were performed in each mouse, with each injury induced upstream of all prior injuries. Any injuries resulting in rupture of the vessel were excluded from the dataset. The dynamic process of platelet accumulation and fibrin formation was analyzed by changes in mean fluorescence intensity over time using SlideBook 6.0 software (Intelligent Imaging Innovations).

**FeCl_3_-induced carotid artery thrombosis assay**

Male and female C57BL/6 WT mice (8 – 10 weeks of age) were designated as either recipient or donor mice. Both groups were treated IP with saline control, CS014 (100 mg/kg), or VPA (100 mg/kg) twice a day for 5 days. On day 6, whole blood was drawn from donor mice to isolate platelets. Due to the high volume of blood needed for platelet isolation, the blood draw from the donor mice is a terminal procedure. The platelets were isolated through gel-filtration using a Sepharose 2B chromatography column and fluorescently labeled with calcein acetoxymethyl (1 μg/mL). Recipient mice were anesthetized by IP injection of ketamine/xylazine and injected intravenously with fluorescently labeled platelets. The mice were placed on a heating pad, and the right common carotid artery was prepared under a dissecting microscope. The mice were then placed on the microscopic stage and thrombus formation was induced via carotid artery injury by topically applying a Whatman paper saturated with 10 % FeCl_3_ for 2 minutes. Following removal of the FeCl_3_, the carotid artery was monitored continuously for platelet adhesion, aggregation and thrombus growth with a Zeiss Axio Examiner Z1 upright fluorescence microscope fitted with a 5X air objective for 30 minutes to determine the time to carotid artery vessel occlusion. Vessel occlusion was defined as time to occlusion from patch placement. Experiments were terminated 30 minutes post-injury due to ethical considerations. All images were recorded and analyzed using SlideBook 6.0 (Intelligent Imaging Innovations).

**Human blood collection and platelet preparation**

All research involving human subjects was carried out in accordance with the Declaration of Helsinki and approved by the University of Michigan Institutional Review Board. Written informed consent was obtained prior to blood collection under the approval of the University of Michigan Institutional Review Board (approval number: HUM00196782). Blood was collected via venipuncture in blue-top BD vacutainers containing 3.2 % sodium citrate (Greiner Bio-One, Monroe, MC). Platelet rich plasma (PRP) was obtained through serial centrifugation of the drawn whole blood for 10 minutes at 200 *g*. Acid citrate dextrose (2.5 % sodium citrate tribasic, 1.5 % citric acid, 2.0 % D-glucose) and apyrase (0.02 U/mL) were added to the PRP prior to centrifugation for 10 minutes at 2000 *g*. Washed platelets were resuspended at a physiological concentration of 3x10^8^ platelet/mL in Tyrode’s buffer (HEPES 10 mM; sodium bicarbonate 12 mM, sodium chloride 127 mM, potassium chloride 5 mM, monosodium phosphate 0.5 mM, magnesium chloride 1 mM, glucose 5 mM, pH 7.4)^3^. Final platelet concentration was determined by a complete blood cell counter (Hemavet 950FS; Drew Scientific, Miami Lakes, FL).

**Platelet aggregation**

Washed human platelets (3x10^8^ platelet/mL) were incubated with the indicated concentrations of VPA, CS014 or control (saline) for 10 minutes at 37^o^C; the compound was added to the platelets and stirred to mix for 2 minutes at 37^o^C prior to removing the platelets from stirring for the remainder of the incubation period. Following incubation, platelets were stimulated with an EC80 concentration of collagen and platelet aggregation was measured for 10 minutes under stirring conditions (1200 rpm) at 37^o^C in a Lumi-Aggregometer (Model 700D, Chrono-log, Havertown, PA). Agonist and treatment concentrations are noted in the figure legend.

**Ex vivo microfluidic perfusion flow chamber**

Microfluidic perfusion chamber slides (µ-slide VI 0.1, ibidi, Martinsried, Germany) were coated with 100 μg/mL collagen type I (Chrono-log, Havertown, PA) and left overnight at 4°C with Tyrode’s buffer. Before use, slides were flushed with Tyrode’s buffer to remove excess collagen. Whole blood was drawn from healthy human donors and collected into 3.2 % sodium citrate vacutainer tubes (Greiner Bio-One, Monroe, NC). Citrated whole blood was incubated with 1 mM or 10 mM of either CS014 or VPA, or the equivalent volume of control (saline) for 10 minutes at 37°C. Following 5 minutes of incubation, platelets were fluorescently labeled by the addition of 2 µM of 3,3’-dihexyloxacarbocyanine iodide (DiOC6; Thermo Fisher Scientific, Waltham, MA). Labeled whole blood was recalcified with 5 mM CaCl_2_ and perfused through the collagen-coated microfluidic chamber heated to 37°C at arterial shear (1800/s) using a syringe pump (Harvard Apparatus, Holliston, MA)^3-5^. Platelet adhesion to the collagen-coated chamber was recorded in real time for 4 minutes under an inverted fluorescent microscope (20X objective Axio Observer Z1 Marianas; Zeiss Industrial Quality Solutions, Wixom, MI). Platelet accumulation was quantified by MFI using SlideBook 6.0 (Intelligent Imaging Innovations, Denver, CO).

**Flow cytometry**

Washed human platelets (3x10^8^ platelet/mL) were treated with increasing concentrations of CS014, VPA or control (saline) for 10 minutes at 37^o^C. Following incubation with the treatment, platelets were stained with a FITC-conjugated antibody specific for the active conformation of the integrin αIIbβ3 (PAC-1), an APC-Cyanine 7-conjugated antibody specific for P-selectin (α-granule secretion marker) and a PE/Dazzle-conjugated antibody specific for CD63 (dense granule secretion marker) (all antibodies purchased from BioLegend, San Diego, CA). Stained platelets were stimulated with the glycoprotein VI (GPVI) receptor agonist convulxin (25 ng/mL) (Cayman Chemical Company, Ann Arbor, MI) in the dark for 10 minutes at 37^o^ C. Convulxin is used as an agonist in flow cytometry because collagen agonist requires shear stress to activate platelets. Following stimulation, platelets were fixed with 2 % paraformaldehyde. The surface expression of activation markers was analyzed by measuring fluorescence intensity using a CytoFLEX flow cytometer (Beckman Coulter, Brea, CA).

**Complete blood count**

Male and female C57BL/6 WT mice (8 – 10 weeks of age) were treated IP with saline control or CS014 (100 mg/kg) twice a day for 5 days. Prior to and following treatment, a complete blood count (CBC) was run using a complete blood cell counter (Hemavet 950FS; Drew Scientific, Miami Lakes, FL). A small volume of blood (~40 µL) was draw from the saphenous vein of the mouse and the whole blood was used to quantify blood cell count.

**Tail bleeding assay**

Male and female C57BL/6 WT mice (8 – 10 weeks of age) were treated IP with saline control, CS014 (100 mg/kg), or VPA (100 mg/kg) twice a day for 5 days. On day 6, mice were anesthetized with ketamine/xylazine delivered via IP injection. Once anesthetized, the mouse was placed on a heating pad in the prone position. The distal 5 mm of the tail was excised with a scalpel blade and the tail was immersed in 37^o^C saline solution. Bleeding time was assessed until cessation of blood flow. Experiments were terminated 10 minutes post tail resection due to ethical considerations.

**Coagulation tests by thromboelastography**

Male and female C57BL/6 WT mice (8 – 10 weeks of age) were treated IP with saline control, CS014 (100 mg/kg), or VPA (100 mg/kg) twice a day for 5 days. On day 6, citrated whole blood was collected with a syringe from the inferior vena cava of anesthetized male and female mice at a 9:1 ratio of blood to sodium citrate (3.8 %). Whole blood from the treated mice (400 μL) was incubated with DMSO control or rivaroxaban (500 ng/ml) for 10 minutes. Treated whole blood (340 mL) was mixed with 20 μL of CaCl_2_ (0.2 M), and viscoelastic properties of whole blood clot formation were studied under low shear stress using the Haemoscope TEG 5000 Thrombelastograph Hemostasis Analyzer (Haemonetics Corp., Braintree, MA) according to the manufacturer’s instructions^6^. Major coagulation parameters including R time (time to initiation of fibrin clot formation), α-angle (the rapidity with which fibrin cross-linking occurs), K time (the time until the clot reaches a strength of 20 mm), maximum amplitude (maximum clot strength), and MRTG (maximum rate of thrombin generation) were analyzed and compared.

**Statistical analysis**

Unpaired two-tailed student *t*-tests, one- and two-way analysis of variance (ANOVA), and two factor mixed-effects model were performed with Prism 9 (GraphPad Software, La Jolla, CA) to analyze the data. Data represent mean ± standard error of the mean (SEM) unless otherwise noted. The specific statistical test used in each experiment is noted in the corresponding figure legend. Determination of statistical significance is indicated where applicable.

CITED LITERATURE

1. Beckman JD, Chen C, Nguyen J, et al. Regulation of heme oxygenase-1 protein expression by miR-377 in combination with miR-217. *J Biol Chem*. Feb 4 2011;286(5):3194-202. doi:10.1074/jbc.M110.148726

2. Falati S, Gross P, Merrill-Skoloff G, Furie BC, Furie B. Real-time in vivo imaging of platelets, tissue factor and fibrin during arterial thrombus formation in the mouse. *Nat Med*. Oct 2002;8(10):1175-81. doi:10.1038/nm782

3. Yamaguchi A, Stanger L, Freedman CJ, et al. DHA 12-LOX-derived oxylipins regulate platelet activation and thrombus formation through a PKA-dependent signaling pathway. *J Thromb Haemost*. Mar 2021;19(3):839-851. doi:10.1111/jth.15184

4. Adili R, Tourdot BE, Mast K, et al. First Selective 12-LOX Inhibitor, ML355, Impairs Thrombus Formation and Vessel Occlusion In Vivo With Minimal Effects on Hemostasis. *Arterioscler Thromb Vasc Biol*. Oct 2017;37(10):1828-1839. doi:10.1161/ATVBAHA.117.309868

5. Stanger L, Yamaguchi A, Yalavarthi P, et al. The oxylipin analog CS585 prevents platelet activation and thrombosis through activation of the prostacyclin receptor. *Blood*. Nov 2 2023;142(18):1556-1569. doi:10.1182/blood.2023020622

6. Kaur H, Fisher K, Othman M. Thromboelastography testing in mice following blood collection from facial vein and cardiac puncture. *Blood Coagul Fibrinolysis*. Oct 2019;30(7):366-369. doi:10.1097/MBC.0000000000000836
